# A Strategy to Deliver Precise Oral Doses of the Glucosinolates or Isothiocyanates from *Moringa oleifera* Leaves for Use in Clinical Studies

**DOI:** 10.3390/nu11071547

**Published:** 2019-07-09

**Authors:** Jed W. Fahey, Kristina L. Wade, Katherine K. Stephenson, Yuzhu Shi, Hua Liu, Anita A. Panjwani, Collin R. Warrick, Mark E. Olson

**Affiliations:** 1 Cullman Chemoprotection Center, Johns Hopkins University, Baltimore, MD 21205, USA; 2 Department of Medicine, Division of Clinical Pharmacology, Johns Hopkins University School of Medicine, Baltimore, MD 21205, USA; 3 Department of Pharmacology and Molecular Sciences, Johns Hopkins University School of Medicine, Baltimore, MD 21205, USA; 4 Department of International Health, Center for Human Nutrition, Johns Hopkins University Bloomberg School of Public Health, Baltimore, MD 21205, USA; 5 Instituto de Biología, Universidad Nacional Autónoma de México, Tercer Circuito de Ciudad Universitaria, Ciuadad de México 04510, Mexico; 6 The International Moringa Germplasm Collection, Jalisco 04510, Mexico

**Keywords:** anti-inflammatory, cytoprotection, drumstick tree, glucomoringin, horseradish tree, indirect antioxidant, leafy vegetable, moringin, phytochemical

## Abstract

The tropical tree *Moringa oleifera* produces high yields of protein-rich leaf biomass, is widely used as a food source, contains an abundance of phytochemicals, and thus has great potential for chronic disease prevention and perhaps, treatment. We have developed and characterized standardized ways of preparing aqueous “teas” from moringa leaves to deliver precisely calibrated levels of phytochemicals for use in clinical trials. These phytochemicals, especially the glucosinolate glucomoringin and the isothiocyanate moringin, produced from it following hydrolysis by the enzyme myrosinase, provide potent anti-inflammatory and cytoprotective indirect antioxidant activity. The taste of both hot and cold teas is palatable without the need for flavor masking. These teas can be easily and reproducibly prepared in underserved tropical regions of the world where moringa is cultivated. Isothiocyanate yield from a cold extraction was rapid and essentially complete after 30 min and its anti-inflammatory potential is comparable to that of equimolar purified moringin. A preparation similar to this may be safe to consume with respect to its bacterial titer even after 48 h without refrigeration. Thus, facile delivery of moringa tea to both adults and children for clinical evaluation of their effects on such conditions as autism, diabetes, and hypertension, is now possible.

## 1. Introduction

*Moringa oleifera,* (common English names include moringa, horseradish tree and drumstick tree) is the most widely cultivated and economically important species of the monogeneric tropical family Moringaceae [1,2,3,4]. Though currently marketed as a “superfood” in the West, it offers many nutritional benefits, as well as medicinal properties that could be of potential benefit to underserved populations in the tropics and sub-tropics [4,5,6]. Human consumption of moringa leaves has been applied to the treatment of conditions such as cancer, regulation of high blood glucose levels, and antibiosis [4], although Western medicine has yet to fully endorse such treatments. These medicinal effects are presumed to be attributable to the characteristic phytochemicals, glucosinolates (GS; primarily glucomoringin) and isothiocyanates (ITC; primarily moringin) found in many parts of the moringa tree and quite prominently in its leaves [4].

**Figure 1:**
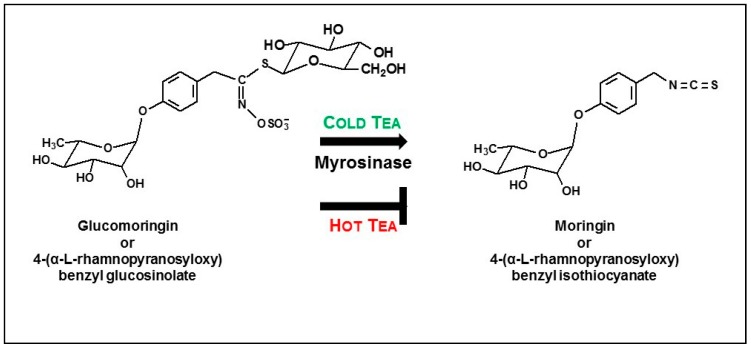
Conversion of glucomoringin to moringin via myrosinase hydrolysis. Glucosinolates such as glucomoringin are converted by the enzyme myrosinase to isothiocyanates such as moringin.

Glucosinolates, also known as mustard oils, are relatively inert, highly water soluble compounds that are characteristic of cruciferous species (the so-called brassica or cruciferous vegetables) including broccoli, radish, cabbage, horseradish, wasabi, and the cresses, as well as a small number of related plant families in the order Brassicales, including Moringaceae [7,8] (Figure 1). Though present at nanomolar concentrations in vacuoles of most tissues in these plants, levels can be as great as almost half-millimolar (26 percent by weight) in the seeds of some plants, including moringa [9]. Once the plant tissues and cells containing these glucosinolates are crushed, broken, torn, chewed, or cut, the GS come in contact with a membrane-bound enzyme called myrosinase (thioglucoside glucohydrolase, E.C. 3.2.1.147), present in many of the same cells that contained those sequestered GS, and enzymatically mediated conversion to ITC happens very rapidly. These ITC are responsible for the harsh/bitter, “horseradishy”, hot, lachrymating sensation caused when consuming many of these plants, and have a variety of well-documented chemoprotective activities [10]. 

Cruciferous vegetables have been far more intensively investigated than have *Moringa* spp. for their chemoprotective properties and for the biological effects of their GS and ITC [11,12]. However, more commonly known cruciferous vegetables are temperate zone crops and are not as readily available in many of the tropical lower- and middle-income countries where the burden of many chronic or non-communicable diseases (NCD) is every bit as high as it is in wealthy countries. In many cases, in particular with autism spectrum disorder (ASD) and schizophrenia, treatment of such psychiatric conditions is generally neither as readily available and accessible nor as sought out (due to stigmatization) [13]. There is a need for alternative/green/frugal medicine—food-based chemoprotection that can be sustainable, inexpensive, and effective [14,15]. Though very few clinical studies have been performed with moringa, several well-done mechanistic studies suggest that it may be effective as primary and/or secondary prevention against an array of chronic conditions including cancer, diabetes, cardiovascular disease, and a number of other neurodegenerative and neurodevelopmental conditions such as ASD and schizophrenia [4]. 

Palatability is a concern that must be overcome in order to encourage adoption of an unfamiliar food in a culture or setting in which it is not normally consumed. The somewhat astringent taste of moringa (some describe it as bitter, harsh, or horseradishy), presents a significant barrier to acceptance, especially in Western cultures not familiar with it. Furthermore, the taste, the bright green color, and the texture and bulk of moringa leaf powder can render it very difficult to mask for blinded clinical studies, especially those with children. One of the ultimate desirable outcomes of this study is to encourage the use of a household-sustainable method of extracting GS and ITC from moringa as a treatment and/or supplement that can be delivered with local foods in clinical studies, for example, of ASD in children. If successful, local treatment could be continued in a cost effective and sustainable manner. 

It is widely known that GS can be readily leached out of plant material or extracted into boiling water along with other beneficial components such as antioxidants [16]. Though the activating enzyme, myrosinase, becomes denatured in this process, the GS remain intact and are not volatile even when subject to reduction. We have created a variety of concentrates, teas, reductions, extracts, and tinctures including alcoholic extracts for addition to a beverage or food of choice with varying impact on the flavor/color/texture of that food. Such approaches are critical for the development of effective phytochemical-targeted dietary interventions to be tested in properly masked randomized controlled trials (RCT). 

Beverages (aqueous extracts hereinafter simply called “teas”) can be readily made, typically from the leaves of glucosinolate-rich plants such as moringa. In addition to taste and palatability, consideration must be given to the desired delivery of potentially bioactive components of the tea—glucomoringin or moringin, respectively, in the case of moringa. Using moringa, a protein-rich edible plant, requires that one must also either consider some form of sterilization or pasteurization of that tea, exploit the innate antimicrobial activity of the tea, or be assured that sources are hygienic and “clean”. Moreover, obtaining and preparing material should be simple and not highly dependent on milling and precise recipes, if the desire is to create a sustainable intervention. 

We have codified the content and availability of the primary phytochemical of interest (glucomoringin), in disease prevention and treatment, in three bulk moringa sources. We have investigated and outlined parameters pertaining to the reproducible production of “herbal” teas from moringa that can be very readily, locally, and sustainably produced for use in clinical trials in the areas where moringa is grown, and for immediate household dietary adoption in those areas.

## 2. Materials and Methods

### 2.1. Sources and Preparation of *Moringa oleifera* Leaf Powder

Three separate sources of *Moringa oleifera* leaf powder were used. These powders will be referred to hereinafter as PA, PB, and PC for the sake of brevity and clarity: PA. This intact leaf material was provided to Johns Hopkins University by Kuli Kuli (Oakland, CA, USA) and was received as a bulk shipment of non-uniform leaflets. These consisted of mostly intact leaflets with some of the finest rachises; most of the thickest rachises were removed and there was considerable breakage of leaflets themselves post-drying, and handling. Harvest date was in 2016 and original provenance was Ghana. A portion of these leaves was milled using a blender (Vitamix Professional Series 750) equipped with a “dry container” filled to approximately one-half of capacity, milled using full power (speed #10) for several ~5 second intervals, stopping and starting so as not to overheat the leaf material and to allow for mixing the leaf material to ensure a homogeneous end product. A total of 1.1 kg of this material was reduced to powder. PB. Moringa leaf powder purchased locally in Baltimore, MD, sold in consumer packaging (a 7.4 oz (210 g) package) in 2018. Provenance was Cambodia (pers. comm. L. Curtis), and label was “Organic Pure Moringa Vegetable Powder, (Kuli Kuli, Oakland, CA, USA, Lot # 20 2012-MLP217067)”. PC. Leaf powder from Moringa Farms, Inc. (Sherman Oaks, CA, USA). This powder (bulk, 1 kg bags) had been stored at 4 °C for approximately 13 years since date of its acquisition, and was grown in California, USA). 

A subset of the powder PA was also sifted, using a graded series of sieves of 2, 1, and 0.5 mm mesh size. The finest of these (0.5 mm maximum particle size) was retained for additional comparisons to the powder (PA) and the leaves from which it was created. 

### 2.2. Extraction of Phytochemicals (Tea Preparation)

Each room temperature aqueous extract (“cold tea”) was prepared by adding 8 g of moringa leaf powder (PA, PB or PC) to 800 mL of ~23 °C deionized water in a glass beaker equipped with a stir-bar and stirred on a magnetic stir-plate for a total of 30 min. Aliquots were taken, vacuum filtered through #41 Whatman filter paper for GS/ITC analysis, immediately, and at 5, 10, 20, and 30 min. Each filtered aliquot was immediately diluted into 100% methanol or the HPLC mobile phase (a solvent mix consisting of 15 mM ammonium formate pH 4.5 in 70:30 (v:v) acetonitrile:water), to halt the reaction of GS and myrosinase, and ITC was measured by cyclocondensation and then by HPLC. Similarly, a boiling water moringa tea (“hot tea”) was prepared by addition of 8 g of leaf powder (PA) to 800 mL of boiling deionized water. Aliquots were sampled and immediately filtered and diluted into HPLC mobile phase as above, for GS/ITC analysis at 0, 1, 3, 5, and 10 min. 

### 2.3. Determination of Glucosinolate (Glucomoringin) Content

HPLC was performed as previously described [17]. Briefly, powders PA, PB, and PC were extracted into a “quadruple” solvent mixture consisting of (1:1:1:1, dimethyl sulfoxide: acetonitrile: dimethyl formamide: water), utilizing high speed tissue homogenization. HPLC, with comparison to pure standards employed a 150 × 4.6 mm, 5 μm particle size, 200 Å pore size column with a silica support and polymeric sulfoalkylbetaine zwitterionic functional groups (ZIC-HILIC; Sequant, Umeå, Sweden). Mobile phase was isocratic 15 mM ammonium formate pH 4.5 in 70:30 (v:v) acetonitrile:water, 0.5 mL/min flow rate, column temperature 25 °C. Detection was by photo diode array with primary monitoring at 235 nm. 

### 2.4. Determination of Isothiocyanate (Moringin) Content by Cyclocondensation

This assay, developed over 25 years ago [18], was conducted essentially as described [19]. Briefly, this assay detects, with equal efficiency, ITC and their dithiocarbamate metabolites. Replicate cyclocondensation reactions consisting of 2 mL; 50% methanol (v/v); 125 mM sodium borate (pH 9.25); 20 mM 1,2-benzenedithiol; and 5% to 25% (v/v) of moringa tea or the organic solvent extract of the powder (PA, PB, and PC) used to make that tea were performed in 4-mL glass vials (2 h; 65 °C). Aliquots of each reaction mixture were injected onto a Whatman Partisil 10 octadecylsilane-2 reverse-phase HPLC column and eluted isocratically with 80% methanol and 20% water (v/v) with detection at 365 nm. The area of the 1,3-benzodithiole-2-thione peak was integrated using Waters Millennium software (version 2.15.01). All samples were assayed at two dilutions, and each analytical run included a sulforaphane standard and the N-acetylcysteine derivative of allyl isothiocyanate as a positive control. 

### 2.5. Myrosinase Activity and Stability

Myrosinase activity in aqueous extracts was analyzed using a chromogenic enzyme assay that employs the hydrolysis of a common and widely available GS, sinigrin (allyl glucosinolate). One unit (1 U) of enzymic activity is defined as that which hydrolyzes 1 μmol of sinigrin per minute in accordance with previously published methods [20,21]. Moringa leaf powders (20 mg/mL) were homogenized in deionized water; daikon myrosinase (used for freeze–thaw stability testing) was in saline solution. Samples to be assessed for enzyme activity were combined with an aqueous solution of 500 μM ascorbic acid and 20 mM sodium phosphate buffer (pH 6.0). Absorbance at 227 nm (A227) was measured as a baseline, sinigrin (50 μM) was added, and rates of sinigrin disappearance were measured by monitoring reduction in A227 (using the molar extinction coefficient ε = 6780 M^−1^cm^−1^ for sinigrin) over 3 min using a SpectraMax Plus plate reader (Molecular Devices, Sunnyvale, CA, USA) and plotting slope of the linear portion of the curve [20,21,22]. 

### 2.6. Soluble Protein Content

Total protein content was determined directly on dilutions of hot moringa tea using the colorimetric bicinchoninic acid or BCA assay in a 96-well microtiter plate format. Absorbance at 562 nm (A562) was measured on a SpectraMax Plus plate reader. Pierce^®^ BCA Reagent and albumin standard from Thermo Scientific (Rockford, IL, USA) were used in accordance with published methods [23]. 

### 2.7. Anti-Inflammatory Activity Assay

The anti-inflammatory activity of moringa teas was gauged by their ability to block the lipopolysaccharide (LPS)-dependent induction of inducible nitric oxide synthase (iNOS) in mouse macrophages. The assay was conducted essentially as described [24]. Briefly, murine macrophage like RAW264.7 cells obtained from the American Type Culture Collection were cultured at 37 °C in DMEM medium containing 10% heat-inactivated fetal bovine serum. Cells were plated in 96-well plates at 20,000 cells per well, grown for 24 h, and exposed to serial dilutions of test reagents in the presence of 10 ng/mL LPS. Cells were incubated at 37 °C in a humidified 5% CO_2_ atmosphere for 48 h. Nitrite concentration in culture supernatants, measured as an indicator of iNOS induction, were determined by the Griess reaction [25]. Nitrite values for cells treated with LPS alone were used as controls. 

### 2.8. Evaluation of Microbiological Load in Moringa Preparations

Aliquots of each of the moringa leaf powders (PA, PB, and PC) were sent to JL Analytical (Modesto, California) for total aerobic bacteria plate count testing. Freshly made cold tea was incubated at ~23 °C, and sampled at 24, 48 and 96 h. for bacterial total aerobic plate count assessments. At each time point, the tea was serially diluted, followed by standard microbial enumeration on Difco Plate Count Agar. 

### 2.9. Reagents and Equipment

All reagents and solvents (Fisher Scientific, Fairlawn, NJ, USA; Sigma-Aldrich, St. Louis, MO, USA; and J. T. Baker, Phillipsburg, NJ, USA) were ACS or HPLC grade. All HPLC components were from Waters (Milford, MA, USA); Model 2695 Alliance System; Model 2996 PDA detectors; Empower software; SpectraMax Plus plate reader (Molecular Devices, Sunnyvale, CA, USA). 

### 2.10. Statistical Analysis

Means, and standard deviations (SD) were computed for replicates (n = 3 unless noted otherwise) for GS, ITC, protein, and myrosinase measurements. For determination of anti-inflammatory activity, the Median Effect Equation of Chou and Talalay was used [26], and computations of *D_m_* were performed using CompuSyn software [27], with *D_m_* representing the concentration at which a 50% effect is obtained. This analysis provides a linear transformation of all experimental observations in the determination of *D_m_*, in contrast to conventional methods of expressing inhibitory potencies (IC50, LD50) which often rely on interpolation between values near the midpoint of effectiveness [28]. Linear regressions, fractional polynomial regressions, and repeated measures ANOVAs were calculated using Stata v. 11.2 (Statacorp, College Station, TX, USA). 

## 3. Results 

### 3.1. Preliminary Analyses of Moringa Leaf Powders for Use in Water Extracts (Teas)

For many years, we have been testing moringa from all over the world, assessing primarily GS content and myrosinase activity, and, to a lesser extent microbial load. We selected three leaf sources representing geographical extremes of source and availability and a wide range of post-harvest storage time, for the comparison of phytochemical-rich extract (tea) suitability. The three moringa leaf sources tested, referred to hereinafter as PA, PB, and PC, were initially assayed for bacterial titer, myrosinase activity, and the predominant and abundant GS in domesticated cultivars of *M. oleifera*, glucomoringin (Table 1) [22].

Myrosinase activities in these powders varied by only two-fold (Table 1). The powder that had been stored for longest period (13 years, PC), had the lowest enzyme activity, but still possessed substantial catalytic activity after this extended storage. 

Glucomoringin levels also varied by only about two-fold (Table 1), but as with myrosinase activity, they were within the central 90 percent of values that we reported in our extensive examination of moringa germplasm [22].

Microbes are omnipresent on fresh fruits and vegetables, yet maintaining low bacterial titer is a main concern when obtaining plant material for human consumption. The American Herbal Products Association suggests microbial levels of less than 10^7^ colony forming units (CFU) per gram for “dried unprocessed herbs for use as ingredients in dietary supplements”, and the United States Pharmacopoeia Convention recommends no more than 10^5^ CFU/g for “dry or powdered botanicals (including tea leaves and other herbal teas)” [29]. The bacterial titer for each of the powders, determined by assessing total aerobic plate count, ranged from 2000 to 24,000 CFU/g (Table 1). Each of these titers was low enough to permit them to be used directly for human consumption.

### 3.2. Glucomoringin and Protein Extraction into Hot Tea

**Figure 2:**
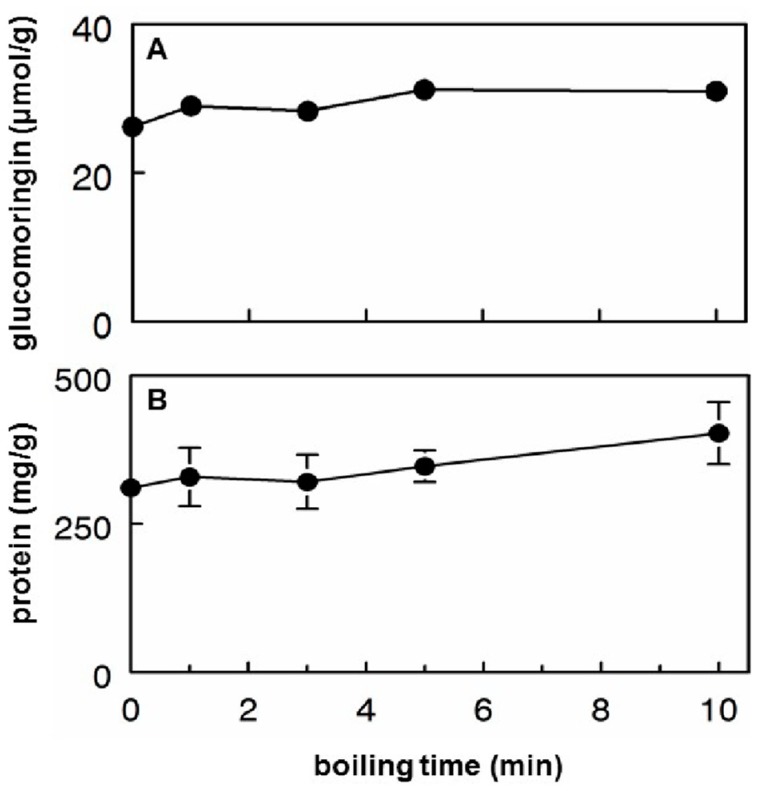
Hot moringa tea prepared with moringa leaf powder PA. (**A**) Glucomoringin extraction was complete within 10 min. (Error bars are obscured by data points). (**B**) Protein also readily dissolved in boiling water. (Error bars are standard deviations (SD); n = 3; some bars are obscured by symbols).

Powder PA was used to perform a timed sampling of hot tea (boiling water extraction) rich in GS. Boiling inactivates myrosinase, preventing the conversion of GS (glucomoringin) to ITC (moringin) (Figure 1). Extraction of glucomoringin occurs rapidly, with a significant effect of boiling time on glucomoringin extraction from powder (F_4,14_ = 10.59, *p* < 0.0028), and extraction is virtually complete within 10 min (Figure 2A), yielding 31 μmol/g, which represents about 40% of the total glucomoringin present in the powder. This estimate is based upon the values obtained from a complete quadruple solvent extraction utilizing high speed tissue homogenization as reported in Table 1. About 400 mg/g total soluble protein was released into the boiling water (Figure 2B), and there was a small, but discernable effect of boiling time on protein extraction (F_4,14_ = 5.12, *p* = 0.0242), which was at the high end of the range we observed in our extensive examination of moringa germplasm [22].

Since release of glucomoringin into hot teas was very rapid (Figure 2A), a range of particle sizes was examined for ease of extraction/release of glucomoringin: (a) the dried leaves originally used to prepare PA, (b) PA, (c) the fine powder obtained after passing PA through a 0.5 mm sieve. There was essentially no difference in glucomoringin extraction among these tea preparations: 28.6 μmol/g for the leaves, 31.0 μmol/g for the PA powder, and 29.7 μmol/g for the finely sieved powder (error for triplicate determinations was less than 5% of the mean in all cases).

### 3.3. Extraction of Moringin into Cold Tea

**Figure 3:**
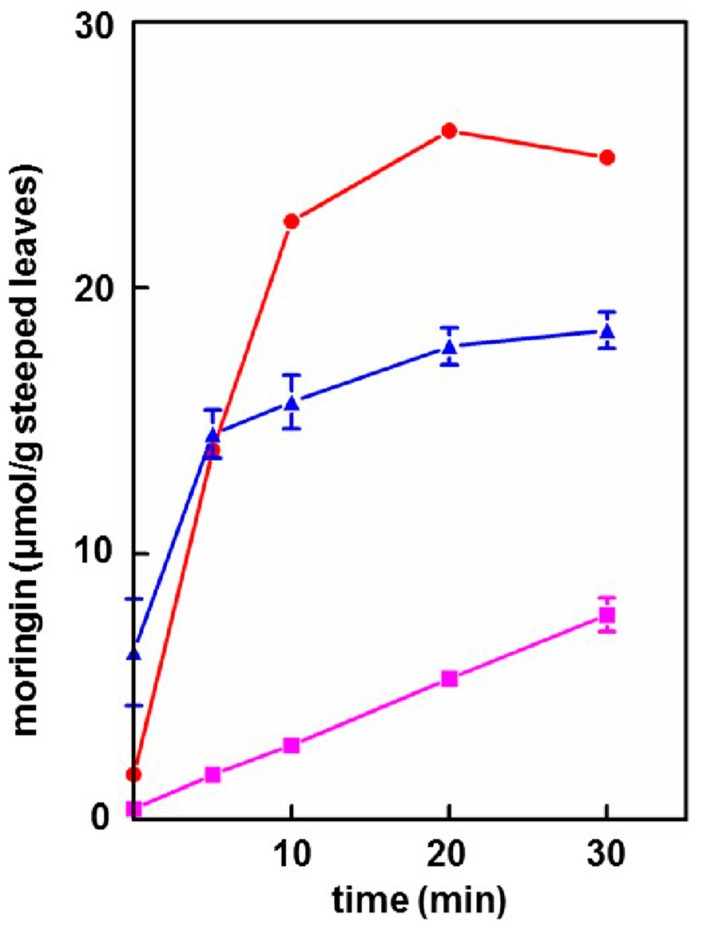
Cold moringa tea prepared with moringa leaf powders PA (●), PB (▲), and PC (■). Morinigin levels in the teas were determined at various time points during room temperature extractions. (Error bars are SD; n = 3; some bars are obscured by symbols).

Endogenous myrosinase in each of the moringa leaf powders was allowed to function as the powders became hydrated at ca. 23 °C, forming the cold teas. Hydrolysis of glucomoringin to moringin (the biologically active ITC) was thus facilitated and moringin yield was estimated by performing the cyclocondensation analysis to measure total ITC (DTC). Maximum levels ranged from 25.9 ± 0.43 μmol/g (PA), to 18.4 ± 0.68 μmol/g (PB), and 7.7 ± 0.64 μmol/g (PC) (Figure 3), which paralleled the ranking of both the myrosinase activities and the glucomoringin levels of the respective powders (Table 1). There was a highly significant effect of incubation time for each powder (*p* < 0.0001), and a significant difference between the moringin yield of powders at 30 min (F_1,6_ = 221.38, *p* < 0.0001). The 30 min incubation (steeping) time reported herein (Figure 3) was based on previous work optimized using the PA powder (data not shown). The powder with the lowest level of myrosinase activity (PC) continued to convert GS to ITC, still increasing in linear fashion at 30 min. At least 25% of the hot water molar yield of glucomoringin was extracted (as moringin) into the cold-brewed tea. 

### 3.4. Microbiological Analysis of Cold Tea Following Non-refrigerated Storage

Hot teas are typically consumed within a short time of being made, and the boiling process reduces the levels of any bacteria associated with the leaf powder to non-detectable levels. Cold teas are not subject to the same sterilizing procedure and we thus assessed bacterial levels (aerobic plate count) in an extract made from PA, incubated at room temperature, and sampled for several days. Plate counts reached a level of only 275 CFU/mL even after 48 h (judged to be a reasonable safe limit for non-refrigerated storage time, and presumed to be a partial consequence of the antimicrobial action of moringin). 

### 3.5. Anti-Inflammatory Activity of Hot and Cold Teas

**Figure 4:**
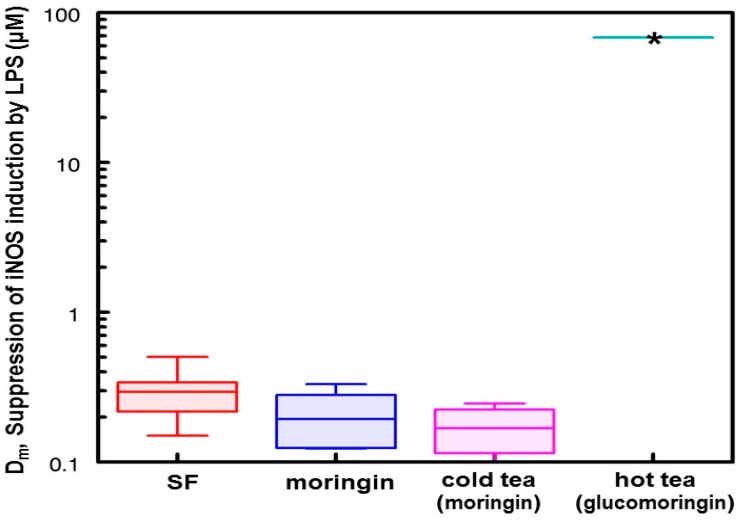
Anti-inflammatory activity in hot and cold moringa teas measured by the inhibition of LPS-stimulated NO production using the Griess assay on mouse macrophage RAW264.7 cells. Results shown are from three separate assays for both cold tea and moringin standard and eight assays for sulforaphane (SF). Boxes show a range between 25th and 75th percentiles; the horizontal line shows the mean value; whiskers extend to the 5th and 95th percentiles. * Activity greater than limit of detection of assay (*D_m_* > 100-fold that of amount of moringin required to achieve a *D_m_*).

Anti-inflammatory activity was measured based on the ability of the teas (as well as component ITC moringin, and sulforaphane (SF) as a well-studied positive control), to suppress (inhibit) LPS-stimulated up-regulation (induction) of iNOS activity using the Griess reaction to quantify NO production in mouse macrophage RAW264.7 cells. The doses required to produce a median effect (*D_m_*) were: SF, 0.29 μM; moringin, 0.19 μM; cold moringa tea, 0.17 μM and hot moringa tea >100 μM (Figure 4). On a molar equivalence basis, the cold tea, containing moringin, had comparable anti-inflammatory activity to the moringin and SF standards used (n = 3 separate assays for both cold tea and moringin standard and n = 8 for SF; F_2,13_ = 2.05, *p* = 0.1751). The hot teas, containing the biologically inert GS, glucomoringin, had no appreciable activity. The amount of tea required to achieve glucomoringin concentration required for detectable inhibition of iNOS activity exceeded the capacity of the assay (plotted as ~100 μM) and was greater than 100-fold more than the amount of moringin required to achieve a *D_m_*.

### 3.6. Myrosinase Stability 

**Figure 5:**
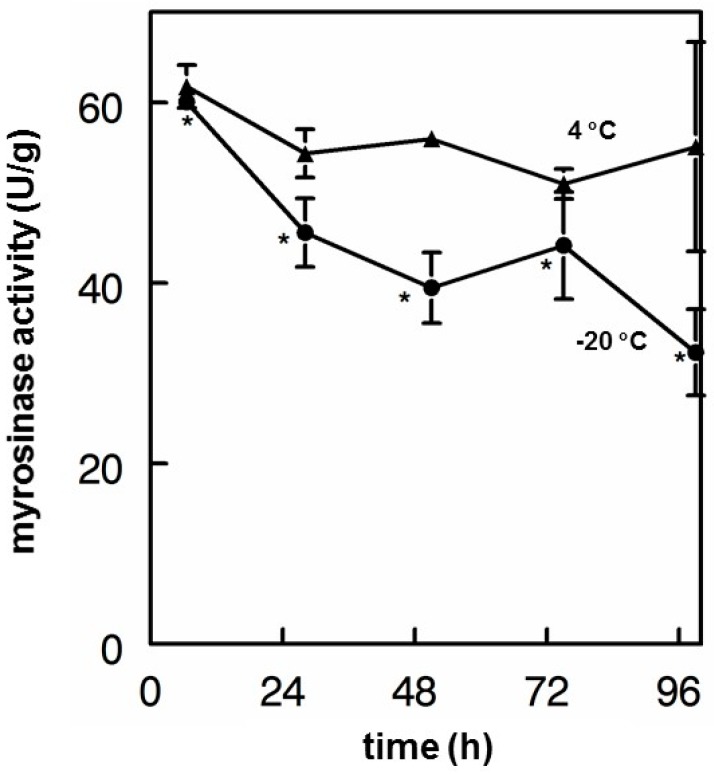
Myrosinase stability in aqueous solution. Daikon myrosinase in saline aqueous solution was stored at 4 °C (▲) or subjected to repeated freeze thaws at –20 °C (●) storage. Each asterisk (*) represents a freeze–thaw; these are cumulative. (Error bars are SD; n = 2).

Myrosinase in solution was tested for its ability to maintain activity for a period of several days under refrigeration and after several freeze–thaw cycles. After four days at 4 °C, myrosinase retained nearly 90% of its initial activity, dropping from 61.8 U/g to 55.1 U/g, but the change was not significant (F_4,9_ = 0.85, *p* = 0.5618). Five repeated freeze–thaw cycles on the same material stored at −20 °C reduced myrosinase activity significantly, from 60.1 U/g to 32.3 U/g (F_4,9_ = 9.44, *p* <0.0257), but without complete loss of activity (Figure 5). 

Similarly, dried myrosinase-rich preparations were evaluated for their stability over a period of about one year at −20 °C, 4 °C, 20 °C, and 50 °C (Supplementary Figure S1). All temperatures were entirely permissive of myrosinase stability except for the 50 °C treatment in which activity declined to about half its initial level after six months, after which it remained fairly constant (4 °C − F_11,35_ = 11.44, *p* < 0.0001; 20 °C − F_11,35_ = 24.06, *p* < 0.0001; −20 °C − F_11,35_ = 4.50, *p* < 0.0013; 50 °C − F_11,35_ = 339, *p* < 0.0001). 

Finally, we examined the longevity of myrosinase in a continually renewed state, i.e., could it be exhausted? Separate experiments using myrosinase purified from broccoli sprouts evaluated the effects of multiple additions of the GS sinigrin (a myrosinase substrate), and multiple additions of ascorbic acid (a co-factor for myrosinase) [21], in a buffered solution, at 4 °C, with repeated sampling. Myrosinase continued hydrolyzing substrate as long as substrate and co-factor were replenished, respectively, as frequently as every 15 min and daily over the course of a four-day incubation (Supplementary Figure S2). 

## 4. Discussion 

We have developed and characterized standardized ways of preparing aqueous “teas” from moringa leaves to deliver precise, calibrated levels of phytochemicals for use in clinical trials. Because bioavailability of ITC (e.g., moringin) from GS (e.g., glucomoringin), when delivered without active myrosinase, are highly variable and are entirely dependent upon the unique gut microbiome of each individual, we explored co-delivery with active myrosinase (e.g., cold tea in which the myrosinase in the moringa leaves is not denatured by steeping in boiling water). 

Our experience preparing and utilizing *Moringa oleifera* (moringa) leaf tea as a source of dietary phytochemicals, and to a lesser extent as a source of protein, extends back for two decades. We previously demonstrated that leaves of moringa contained about 30% protein on a dry weight basis, yielding about 25% soluble protein [3]. In subsequent work, domesticated cultivars of moringa averaged about 30% protein by weight, 75 μmol glucomoringin per gram of leaf powder, 26 U of myrosinase per gram, and an antioxidant (ABTS) ED50 of about 30 units/g [22]. All of these rough averages, of course, came with great cultivar- and provenance-based variability, but by and large it told us that a single cup of tea (8 ounces) made from moringa leaves could be expected to yield only about 0.6 g of protein to the consumer. This is not very impressive from the perspective of dietary protein requirements that range upwards from about 0.8 g protein per kg body weight per day [30]. Much more interesting was the fact that this cup of tea could be expected to deliver about 200 μmol of glucomoringin, a theoretically therapeutic and/or preventive dose in clinical settings, based upon our extensive previous investigations of these compounds [31] and our multiple clinical trials in China with glucoraphanin (a GS) and its cognate ITC SF [32,33], very close phytochemical relatives of glucomoringin and moringin, respectively [7,31]. 

Furthermore, we previously demonstrated the ability of moringa to induce cytoprotective and antioxidant enzymes and to elevate glutathione (GSH) levels in a variety of cell lines [31]. This, as well as its relatively high direct antioxidant activity, encouraged us to evaluate herein, the effect of moringin and of a tea rich in moringin (produced by myrosinase catalyzed hydrolysis of glucomoringin), on suppression of LPS-stimulated transcriptional activation of iNOS, by measuring NO production using the Griess reaction in RAW264.7 macrophages. This *in vitro* anti-inflammatory assay nicely complements the ABTS assessment of Trolox equivalent antioxidant capacity, which we have utilized with moringin [22] and other “direct” antioxidant phytochemicals [34]. 

The active myrosinase presence in these leaves allows us to predict rapid and complete conversion of glucomoringin to moringin (with antibacterial potency) at ambient temperatures [35], in a time frame that could preclude microbial overgrowth, even should the leaves used for to make tea be heavily contaminated with an environmental microbe inoculum. Furthermore, our previous work in Mexico [3,22,31], in which teas were made from moringa as a means of extracting bioactives, led us to believe that the innate bacteriostatic and fungistatic activity of these teas due to the antibiosis of moringin [36,37] might lend them to daily or even less frequent preparation without concomitant risk of microbial overgrowth. 

We thus chose three very different sources of moringa leaf powder with which to replicate our results, and showed that regardless of source:

(A) About half of the available glucomoringin (the highly water soluble but biologically inactive precursor to the chemoprotective ITC) could be rapidly extracted into a boiling water tea made from the leaves. This yield was almost complete in that a tea sample removed immediately upon placing powder into boiling water contained essentially all the glucomoringin that could be extracted even with much longer boiling times (up to 30 min; data not shown). The comparator in all cases was a quadruple organic solvent extraction (water, acetonitrile, dimethyl sulfoxide and dimethyl fumarate, in equal portions) in which the powder was thoroughly extracted using a mechanical tissue homogenizer.
(B) Particle size did not matter, so that using a finely ground and sieved powder yielded no more glucomoringin than using coarsely chopped leaves. This of course has implications for making tea infusions from dried (whole or coarsely chopped) moringa leaflets because it is much easier to filter, and requires less processing. That the fineness of milling is not a critical factor influencing glucomoringin yield means that relatively comparable results can be achieved across studies in different parts of the world.
(C) Provided that the powder contained active myrosinase, a cold water (23 °C) tea in which the powder incubated, or steeped, to produce an infusion, yielded slightly less of the cognate ITC (on a molar basis) than the GS extracted into a comparable boiling water tea. Yield from leaf powders with highly active myrosinase and high glucomoringin content plateaued within about 10 min, whereas yield from powder with less myrosinase kept increasing even after 30 min. The fact that moringin yield from cold teas made from PA and PB plateau within the 30 min incubation time, indicated completed extraction and hydrolysis, whereas the linear response at 30 min from PC indicated that it may benefit from a longer incubation time.
(D) Bacterial titer in hot teas was not a concern with imminent consumption, whereas titer (aerobic plate count) in cold teas that had been sitting at room temperature for 48 h was still perfectly acceptable. (A limit of 10^5^ CFU/mL is recommended by the United States Pharmacopoeia Convention for dried or powdered botanicals and a limit of 10^7^ CFU/g is recommended by the International Standard/American National Standard for Dietary Supplements (NSF/ANSI), [29]). These low bacterial counts were most likely due to the bacteriostatic activity of the moringin that was produced in the tea [36,37].
(E) Based on the number of μmol of moringin or glucomoringin in the teas, moringin-containing tea was as potent as pure moringin in inhibiting LPS-stimulated NO production, which is an anti-inflammatory response indicator. Pure moringin was even more potent, on a molar basis, than sulforaphane from broccoli sprouts. Conversely, the glucomoringin-containing boiled tea had no inherent anti-inflammatory activity, though it is presumed that upon ingestion and digestion (the gut microflora contains some myrosinase activity), some moringin would be produced and absorbed.
(F) Taste of both hot and cold teas was judged to be highly acceptable, by casual survey of friends and family who tasted them. Further larger studies are warranted to confirm this finding in the general population and the ASD population. Moringa tea is widely sold commercially, showing that it already has some acceptance worldwide. We have thus abandoned earlier effort to develop taste masking strategies for delivery of dried leaf powder to children with ASD. This was something we had regarded as a necessary prerequisite for delivery in a clinical study to children who characteristically have very strong taste aversions or preferences, and who by-and-large do not, will not, or cannot take the pills (capsules or tablets) that would be required to deliver a sufficient quantity of leaf powder to provide enough glucormoringin (e.g., 200 μmol or more) for the trials that we envisioned. The favorable taste profile combined with the extraordinarily low bacterial plate count of these teas may be useful for people interested in the preparation of cold (ITC-rich) moringa leaf powder teas that we feel will have the greatest chemoprotection potential.
(G) Finally, myrosinase isolated and purified from moringa and cruciferous vegetables has historically been viewed as being highly unstable. We found this not to be the case, showing that enzyme in saline solution was quite stable for years, and that these solutions could be repeatedly frozen and thawed but could also be refrigerated for multiple days without substantial attenuation of activity. Moreover, we have determined that dry moringa leaf powder (e.g., “PC”) could be stored at 4 °C for 13 years and retain enough myrosinase activity to make a potent cold tea, hydrolyzing glucomoringin to moringin.

Somewhat surprising, and of great value to the ultimate objectives of this study (developing preparations for both clinical trials and household preparation), were the findings that: ITC yield from a cold (room temperature) extraction was rapid and essentially complete after 30 min and that a preparation similar to this may be safe to consume with respect to its bacterial titer even after 48 h without refrigeration. Moreover, cold-brewed moringa tea was highly anti-inflammatory and all of the anti-inflammatory activity could be accounted for by the moringin present in those extracts. Hot-brewed moringa teas are now widely available to consumers and have achieved acceptance in Western markets, though they tend towards astringency. Although cold-brewed teas contain isothiocyanates, we presume that the reduced extraction of various tannins and phenolic astringent compounds into cold water, led to the somewhat milder and more pleasing taste in these teas.

We and others have suggested that the unique rhamnopyranosyloxy(benzyl) functional group on the molecule leads not only to its unique properties, but to its enhanced stability among ITC, when extracted from the plant [7,31,38]. Thus, we encourage the use of cold water extracts of moringa leaves or leaf powder, to be used in clinical interventions to evaluate any of a wide range of conditions for which traditional medicine and modern laboratory and pre-clinical studies suggest biologic plausibility.

## Figures and Tables

**Table 1:** Characteristics of moringa leaf powders used in this study.

Moringa Leaf Powder	Bacterial Titer ^c^ (CFU/g)	Myrosinase Activity ^d^ (U/g)	Glucomoringin Content ^d,e^ (µmol/g)*
PA ^a^	2000	11.56 ± 0.27	77.03 ± 6.52
PB ^b^	24,000	8.71 ± 0.09	49.65 ± 4.58
PC ^b^	2000	6.25 ± 1.61	40.25 ± 2.19

^a^ n = 3; ^b^ n = 2; ^c^ A single, multi-gram batch was sent for independent microbial testing at a commercial lab (see Methods); CFU/g—colony forming units/gram ^d^ Values given are Units/gram ± standard deviation (SD); ^e,^* Assessed in extracts prepared with tissue homegenizer, in 1:1:1:1 water:acetonitrile:dimethyl sulfoxide:dimethyl fumarate.

## Supplementary Materials

The following are available online at https://www.mdpi.com/2072-6643/11/7/1547/s1, Figure S1: Effect of storage temperature on myrosinase activity. Ground broccoli seed meal was stored at −20 °C (▲), 4 °C (●), 20 °C (♦), and 50 °C (■) and the myrosinase activity tested at intervals over almost 400 days. There was almost no reduction in myrosinase activity at any of the storage temperatures except 50 °C, at which activity was approximately halved within the first 6 months and stable thereafter. (Error bars are SD; *n* = 3), Figure S2: Sustained activity of myrosinase in a representative experiment. Myrosinase is an enzyme that will continue to function as long as it has an optimal (but not inhibitory) concentration of its obligate co-factor, ascorbic acid, and adequate substrate (sinigrin, in this case). The reaction diagrammed took place in a glass scintillation vial that was “refreshed” with co-factor and substrate as indicated on the graph and monitored for completion by HPLC [31]. At top: Ascorbic acid (**↓**, in blue) added to final concentration of 500 µM; Repeated 1 µmol additions of sinigrin (**↓**, in red), with each spike representing a further addition; Myrosinase “failure” (**↓, in black**)—each added 1 µmol aliquot of sinigrin that is not fully hydrolyzed starts to build up, signaling reduced enzyme catalysis; Overnight incubation (**↓**, in green) at 4 °C, or otherwise incubation was at 25 °C.
