# Supplementary material for: A Strategy to Deliver Precise Oral Doses of the Glucosinolates or Isothiocyanates from Moringa oleifera Leaves for Use in Clinical Studies
Source: Nutrients. 2019 Jul 9;11(7):1547. doi: 10.3390/nu11071547 (PMC6682957; doi:10.3390/nu11071547)
Supplement: Supplementary file 1 [file nutrients-11-01547-s001.pdf]

Supplementary Information for:

A Strategy to Deliver Precise Oral Doses of the Glucosinolates or Isothiocyanates from *Moringa oleifera* Leaves for Use in Clinical Studies

Jed W. Fahey[1,2,3,4 *], Kristina L. Wade[1,3], Katherine K. Stephenson[1,3],Yuzhu Shi[1], Hua Liu[1,3], Anita A. Panjwani[1,4], Collin R. Warrick[1,3], Mark E. Olson[5,6]

[1] *Cullman Chemoprotection Center, Johns Hopkins University, Baltimore, Maryland, 21205 USA*

[2] *Johns Hopkins University School of Medicine, Department of Medicine, Division of Clinical Pharmacology, Baltimore, Maryland, 21205 USA*

[3] *Johns Hopkins University School of Medicine, Department of Pharmacology and Molecular Sciences, Baltimore, Maryland, 21205 USA*

[4] *Johns Hopkins University Bloomberg School of Public Health, Department of International Health, Center for Human Nutrition, Baltimore, Maryland, 21205 USA*

[5] *Instituto de Biología, Universidad Nacional Autónoma de México, Tercer Circuito de Ciudad Universitaria, Ciuadad de México 04510, Mexico*

[6] *The International Moringa Germplasm Collection, Jalisco, Mexico*

*Address for Correspondence: Dr. Jed W. Fahey, Cullman Chemoprotection Center, 855 N. Wolfe St., Suite 625, Baltimore, Maryland, USA 21205. (jfahey@jhmi.edu)

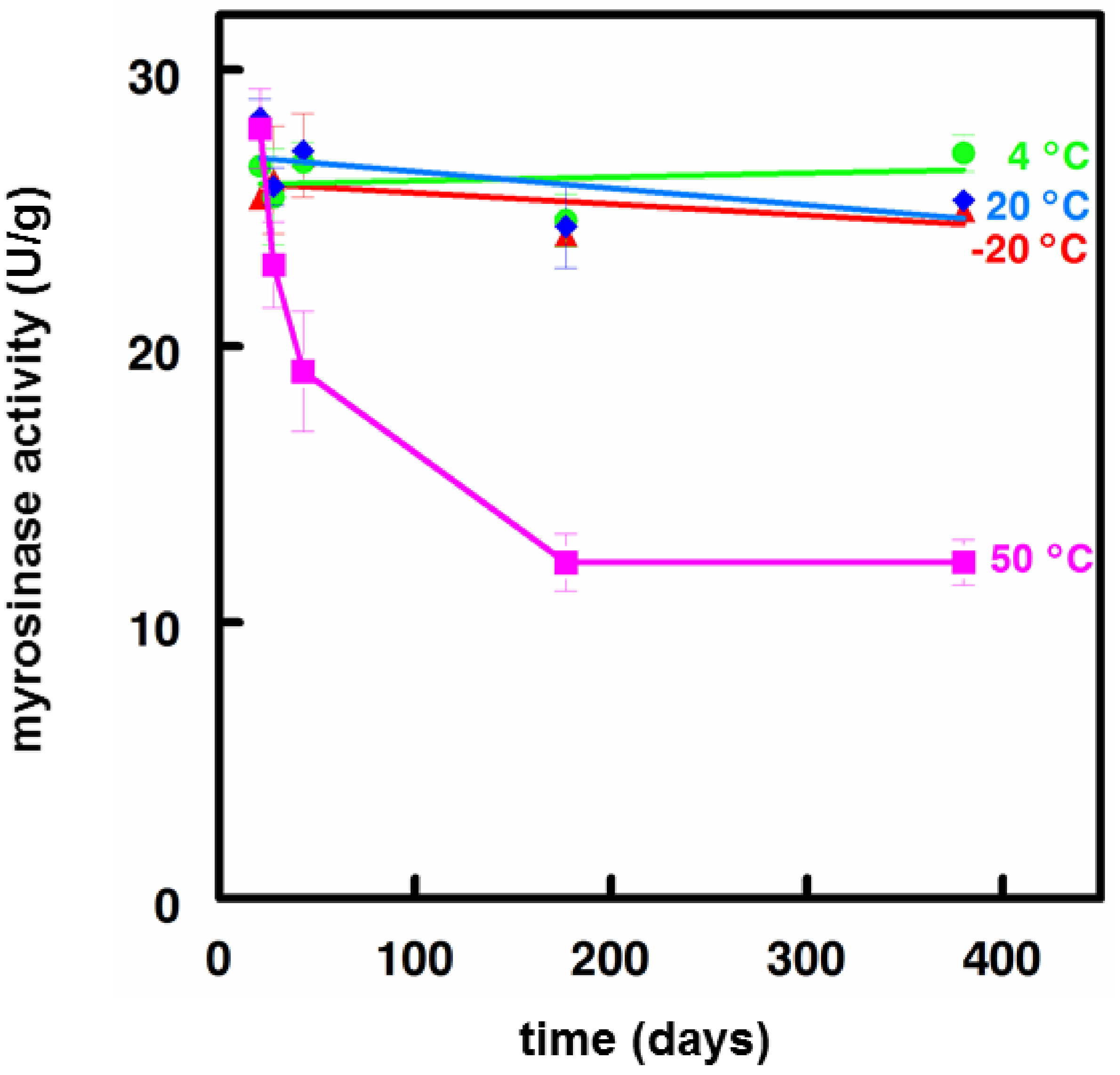

Supplemental Figure S1. Effect of storage temperature on myrosinase activity. Ground broccoli seed meal was stored at -20 ℃ (▲), 4 ℃ (●), 20 ℃ (♦), and 50 ℃ (■) and the myrosinase activity tested at intervals over almost 400 days. There was almost no reduction in myrosinase activity at any of the storage temperatures except 50 ºC, at which activity was approximately halved within the first 6 months and stable thereafter. (Error bars are SD; n = 3).

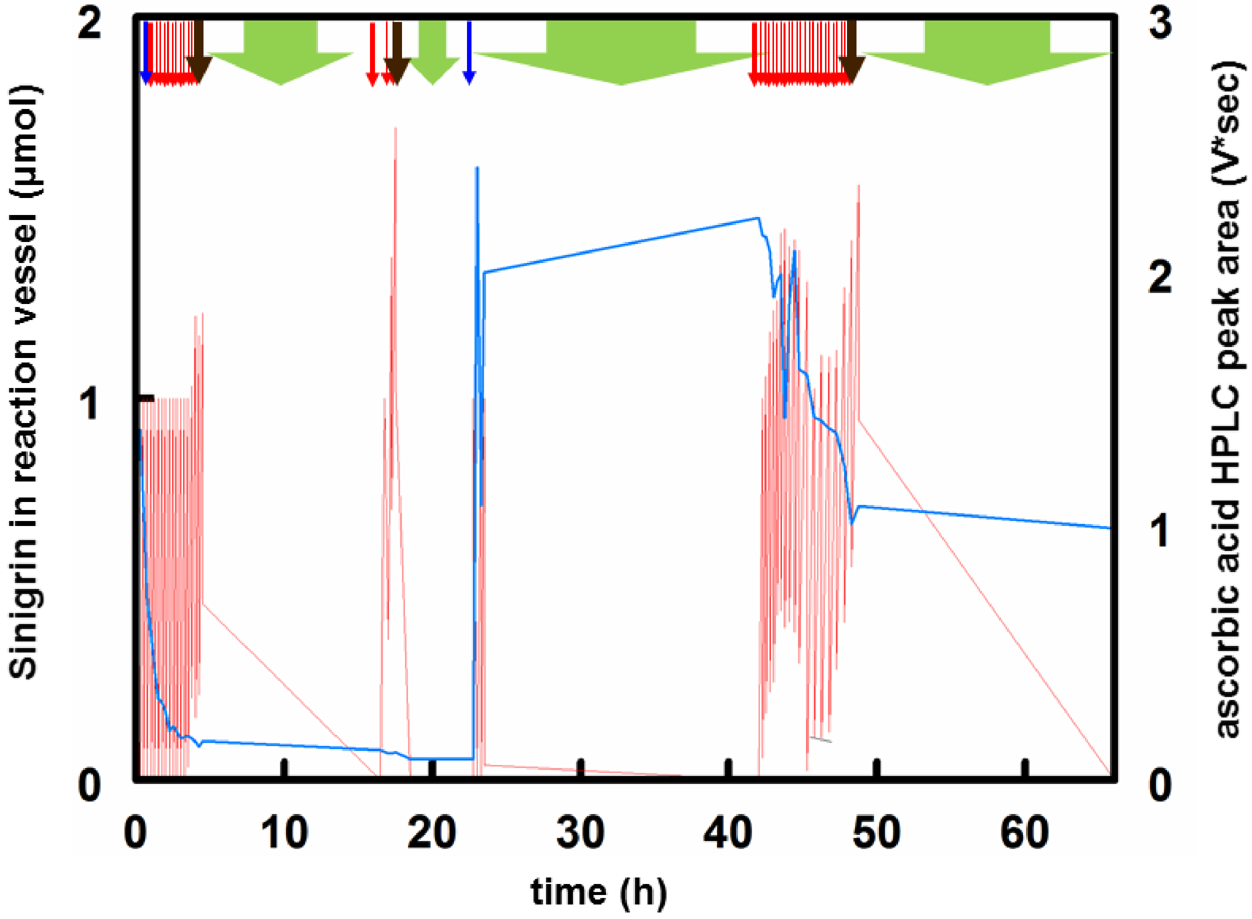

Supplemental Figure S2. Sustained activity of myrosinase in a representative experiment. Myrosinase is an enzyme that will continue to function as long as it has an optimal (but not inhibitory) concentration of its obligate co-factor, ascorbic acid, and adequate substrate (sinigrin, in this case). The reaction diagrammed took place in a glass scintillation vial that was "refreshed" with co-factor and substrate as indicated on the graph and monitored for completion by HPLC [31]. *At top:* Ascorbic acid (↓, in blue) added to final concentration of 500 µM; Repeated 1 µmol additions of sinigrin (↓, in red), with each spike representing a further addition; Myrosinase "failure" (↓, in black) – each added 1 µmol aliquot of sinigrin that is not fully hydrolyzed starts to build up, signaling reduced enzyme catalysis; Overnight incubation (↓, in green) at 4 °C, or otherwise incubation was at 25 °C.
